# MAPK Pathway under Chronic Copper Excess in Green Macroalgae (Chlorophyta): Influence on Metal Exclusion/Extrusion Mechanisms and Photosynthesis

**DOI:** 10.3390/ijms20184547

**Published:** 2019-09-13

**Authors:** Paula S. M. Celis-Plá, Fernanda Rodríguez-Rojas, Lorena Méndez, Fabiola Moenne, Pamela T. Muñoz, M. Gabriela Lobos, Patricia Díaz, José Luis Sánchez-Lizaso, Murray T. Brown, Alejandra Moenne, Claudio A. Sáez

**Affiliations:** 1 Laboratory of Aquatic Environmental Research, Centro de Estudios Avanzados, Universidad de Playa Ancha, Viña del Mar 2520000, Chile; paulacelispla@upla.cl (P.S.M.C.-P.); fernanda.rodriguez@upla.cl (F.R.-R.); lorena.mendez@alumnos.uv.cl (L.M.); fabiola.moenne@upla.cl (F.M.); pamela.munoz@upla.cl (P.T.M.); 2 Doctorado Interdisciplinario en Ciencias Ambientales, Facultad de Ciencias Naturales y Exactas, Universidad de Playa Ancha, Valparaíso 2340000, Chile; 3 Doctorado en Ciencias del Mar y Biología Aplicada, Departamento de Ciencias del Mar y Biología Aplicada, Universidad de Alicante, 03080 Alicante, Spain; 4 Laboratory of Environmental and Analytical Chemistry, Instituto de Química y Bioquímica, Facultad de Ciencias, Universidad de Valparaíso, Valparaíso 234000, Chile; gabriela.lobos@uv.cl (M.G.L.); patriciaediazg@gmail.com (P.D.); 5 Departamento de Ciencias del Mar y Biología Aplicada, Universidad de Alicante, 03080 Alicante, Spain; jl.sanchez@ua.es; 6 School of Biological and Marine Sciences, University of Plymouth, Plymouth PL4 8AA, UK; mt.brown@plymouth.ac.uk; 7 Laboratory of Marine Biotechnology, Facultad de Química y Biología, Universidad de Santiago de Chile, Santiago 9170020, Chile; alejandra.moenne@usach.cl; 8 HUB-AMBIENTAL UPLA, Universidad de Playa Ancha, Valparaíso 2340000, Chile

**Keywords:** in vivo chlorophyll *a* florescence, physiology, mitogen activated protein kinases, *Ulva compressa*, metal accumulation

## Abstract

There is currently no information regarding the role that whole mitogen activated protein kinase (MAPK) pathways play in counteracting environmental stress in photosynthetic organisms. To address this gap, we exposed *Ulva compressa* to chronic levels of copper (10 µM) specific inhibitors of Extracellular Signal Regulated Kinases (ERK), c-Jun *N*-terminal Kinases (JNK), and Cytokinin Specific Binding Protein (p38) MAPKs alone or in combination. Intracellular copper accumulation and photosynthetic activity (in vivo chlorophyll *a* fluorescence) were measured after 6 h, 24 h, 48 h, and 6 days of exposure. By day 6, when one (except JNK) or more of the MAPK pathways were inhibited under copper stress, there was a decrease in copper accumulation compared with algae exposed to copper alone. When at least two MAPKs were blocked, there was a decrease in photosynthetic activity expressed in lower productivity (ETR_max_), efficiency (α_ETR_), and saturation of irradiance (EkETR), accompanied by higher non-photochemical quenching (NPQ_max_), compared to both the control and copper-only treatments. In terms of accumulation, once the MAPK pathways were partially or completely blocked under copper, there was crosstalk between these and other signaling mechanisms to enhance metal extrusion/exclusion from cells. Crosstalk occurred among MAPK pathways to maintain photosynthesis homeostasis, demonstrating the importance of the signaling pathways for physiological performance. This study is complemented by a parallel/complementary article Rodríguez-Rojas et al. on the role of MAPKs in copper-detoxification.

## 1. Introduction

In eukaryotes, including mammals, plants, and algae, stimuli such as reactive oxygen species (ROS), growth factors, cytokines, and metal excess can activate mitogen activated protein kinases (MAPKs). These enzymes participate in an important cellular signaling pathway which regulates metabolic and physiological processes, including transcription, mitosis, and cell differentiation, in response to biotic and environmental stressors [1,2,3]. MAPKs are serine/threonine kinases that are activated by a sequenced phosphorylation cascade involving MAPK kinases (MAPKK), which are induced upstream by MAPKK kinases (MAPKKK). There are three main MAPK pathways, which correspond to those leading to MAPK Extracellular Signal Regulated Kinases (ERK), c-Jun *N*-terminal Kinases (JNK), and Cytokinin Specific Binding Protein (p38) [2,3]. The exact mechanism by which ROS activate MAPK pathways is still not well understood, but it has been established that the accumulation of ROS, such as superoxide anions, hydrogen peroxide and hydroxyl radicals, oxidizes thioredoxins (TRX) and binding apoptosis stimulating kinase 1 (ASK1), leading to the dissociation of TRX from ASK1; the latter has been shown to trigger at least the JNK and p38 MAPK pathways [4]. In addition, cysteine residues present in the receptors of cytokines and growth factors are oxidized by ROS leading to their activation, as evidenced by antioxidants inhibiting EGF-1-induced ROS production and activation of the ERK pathway [5]. Furthermore, hydrogen peroxide (H_2_O_2_) directly induces growth factor receptors and MAPK pathways in different cell types [6]. A potential alternative mechanism is the direct ROS-oxidation and degradation of phosphatases that inhibit MAPK pathways [7]. Another factor known to activate MAPKs is excess cellular metal concentrations, although it is generally agreed that the mechanisms by which metals trigger the pathway relate to metal-mediated overproduction of ROS [8]. For example, copper can induce ROS by substituting iron in a Fenton-like reaction, disrupting electron transport chains in chloroplasts and mitochondria, and facilitating excess energy transfer to oxygen [9,10]. The signaling role of ROS is well-known; however, beyond certain threshold levels there can be an imbalance in reactive oxygen metabolism resulting in oxidative stress and, in severe cases, damage [9].

The biological implications of copper excess are well-documented in photoautotrophs from terrestrial and aquatic ecosystems [11,12,13,14]. Due to domestic and industrial activities, copper is recognized as one of the most pernicious pollutants in the marine environment [15]. As well as their role as primary producers, marine macroalgae or seaweeds are regarded as significant habitat-forming organisms, and the detrimental effects of copper pollution under both laboratory and field assessments have been explored in some detail [12,16,17,18]. The main recognized biological strategies in macroalgae to counteract copper (and other metals) excess are related to cellular exclusion/extrusion mechanisms, detoxification through the syntheses of metal chelators, and activation of antioxidant defences. Most of these processes were observed to be transcriptionally regulated [9,18,19]. Despite this, there is a paucity of information on signal transduction pathways involved in metal tolerance. Records in other photoautotrophs indicate that the main pathways underlying metal tolerance are associated with ROS, nitric oxide (NO), MAPKs, hormones, and calcium signaling [8]. Of these, the best described for macroalgae are the calcium, NO, and ROS signaling mechanisms, using the copper-tolerant green macroalga *Ulva compressa* as a model species [9]. Under chronic copper exposure of 10 µM, *U. compressa* internalizes calcium from the extracellular media mediated by transient dependent potential (TRP) and voltage-gated calcium channels (VDCC), processes that induce calcium release from the endoplasmic reticulum (ER). This, in turn, induces calcium crosstalk with ROS and NO, the activation of calmodulins (CaMs) and calcium-dependent protein kinases (CDPKs), and the subsequent upregulation of enzymes involved in ROS detoxification [20,21,22,23,24]. Recent findings also suggest that the MAPK ERK pathway may play a role in the regulation of antioxidant enzymes and in the synthesis of metallothioneins (MTs), which are metal chelators [20]. However, the dynamics and overall contributions of the ERK and MAPK pathways in regulating metal-tolerance mechanisms in macroalgae remain unclear.

Once intracellular copper concentrations reach toxic levels, biological impairment manifests itself in physiological processes, with photosynthetic activity being an important example [25]. A reliable, non-invasive technique for the measurement of photosynthesis is pulse amplitude modulation (PAM) chlorophyll *a* fluorescence associated with photosystem II (PSII), which is used as an indicator of eco-physiological performance [26,27]. Information on various functional components of the photosynthetic apparatus is acquired, including the maximal quantum yield of PSII (*F_v_/F_m_*), which implies the state of photoinhibition [28], the maximal electron transport rate (ETR_max_), the photosynthetic efficiency (α_ETR_), and the saturation of irradiance (EkETR), which estimate photosynthetic capacity, efficiency, and productivity, and non-photochemical quenching (NPQ), which is considered to be an indicator of photoprotection that is accomplished through xanthophyll cycle-mediated energy dissipation [12,29]. Several published studies on the effects of high copper levels on photosynthetic activity in *Ulva* spp. report a decrease in *F_v_/F_m_* and rETR [30] and high NPQ [31]. These patterns are indicative of copper-induced overstimulation of electron transport chains, triggering decreased photosynthetic activity, and the activation of energy dissipation mechanisms to avoid damage in PSII. As with other photoautotrophs, no records have been published regarding the involvement of the MAPK signaling pathway in photosynthesis in macroalgae.

In this investigation, we studied the roles of the three main pathways within the MAPK signaling cascade on the physiological processes associated with copper accumulation and photosynthetic activity in green macroalgae, using *U. compressa* as a model organism. Specific inhibitors of the ERK, JNK, and p38 pathways were applied alone or in combination under copper excess. The implications for metal extrusion/exclusion mechanisms and photosynthetic activity, as determined from measurements of *F_v_/F_m_*, ETR_max_, α_ETR_, Ek_ETR_, and NPQ_max_, were evaluated. In a parallel study by Rodríguez-Rojas et al. [1], we extended this research to assess the role of the MAPK pathway in the toxicity/detoxification mechanisms of *U. compressa* under copper excess.

## 2. Results

### 2.1. Copper Accumulation

**Figure 1:**
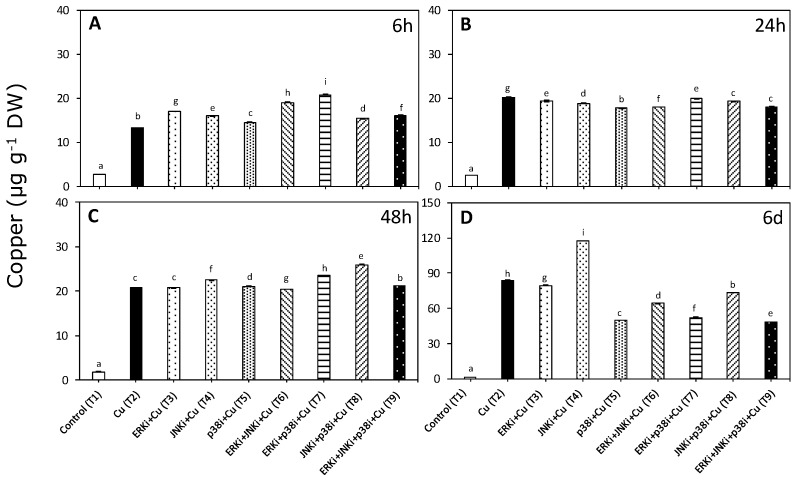
Intracellular copper accumulation in *Ulva compressa* under copper and/or exposure to mitogen activated protein kinase (MAPK) inhibitors. Treatments: T1—control only with seawater; T2—copper only exposure as 10 µM CuSO_4_ (Cu); T3—copper + 5 µM MAPK Extracellular Signal Regulated Kinase (ERK) inhibitor PD98059 (Cu + ERKi); T4—copper + 5 µM MAPK c-Jun *N*-terminal Kinase (JNK) inhibitor SP600125 (Cu + JNKi); T5—copper + MAPK Cytokinin Specific Binding Protein (p38) inhibitor SB203580 (Cu + p38i); T6—Cu + ERKi + JNKi; T7—Cu + ERKi + p38i; T8—Cu + p38i + JNKi; and T9—Cu + ERKi + JNKi + p38i. Samples were analyzed after (**A**) 6 h, (**B**) 24 h, (**C**) 48 h, and (**D**) 6 days. Different letters within each graph represent significant differences at the 95% confidence interval (*p* < 0.05). Plots are mean ± SE (*n* = 3).

Copper accumulation was significantly higher than in controls when *U. compressa* was exposed to copper, although the patterns differed between treatments in the presence of MAPK inhibitors and at different times (*p* < 0.01, Figure 1, Table S1). Average intracellular concentrations were around 81% of the total accumulated copper (intracellular plus extracellular).

Copper accumulation at 6 h was highest in treatments containing copper and ERK inhibitor (T3, T6, T7, and T9) and JNK (T4) or p38 (T5) inhibitors. Moreover, seaweeds exposed to all treatments with copper and inhibitors (T3–T9) had higher intracellular copper levels that those exposed to only copper (Figure 1A). In contrast, at 24 h, the copper content was always lower in treatments containing copper and inhibitors compared with only copper (T3–T9). Among treatments with copper and inhibitors, the lowest levels of accumulation were detected when JNKi, p38i, and ERK were applied together (T9) (Figure 1B). Patterns at 48 h were similar to those observed at 6 h, although the most accumulation was recorded in treatments with copper and p38 inhibitor (T5, T7, T8, and T9) and with either ERK (T3) or JNK inhibitors (T4) (Figure 1C). For all treatments, the accumulation of copper was highest after exposure for 6 d (Figure 1A–D). Following the oscillatory trends found throughout the experiment, except for copper with JNK inhibitor (T4), at day 6, the metal accumulation levels in treatments combining copper and inhibitors (T3–T9) were significantly lower than with copper alone (T2) (Figure 1D). Intracellular copper concentrations in treatments with copper and inhibitors were as follows: ERK and p38 (T7) < p38 (T5) < ERK < p38 and JNK (T9) (Figure 1D).

### 2.2. Photosynthetic Activity According to In Vivo Chlorophyll a Florescence

**Figure 2:**
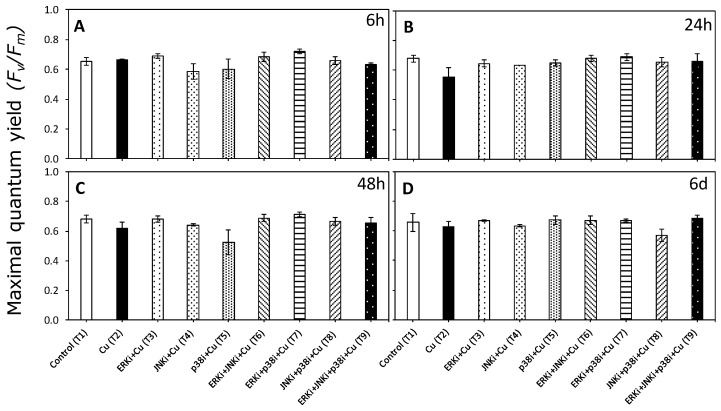
Maximum quantum yield of photosystem II (PSII) (*F_v_/F_m_*) in *U. compressa* under copper and/or exposure to MAPK inhibitors. Treatments: T1—control only with seawater; T2—copper only exposure as 10 µM CuSO_4_ (Cu); T3—copper + 5 µM MAPK ERK inhibitor PD98059 (Cu + ERKi); T4—copper + 5 µM MAPK JNK inhibitor SP600125 (Cu + JNKi); T5—copper + MAPK p38 inhibitor SB203580 (Cu + p38i); T6—Cu + ERKi + JNKi; T7—Cu + ERKi + p38i; T8—Cu + p38i + JNKi; and T9—Cu + ERKi + JNKi + p38i. Samples were analyzed after (**A**) 6 h, (**B**) 24 h, (**C**) 48 h, and (**D**) 6 d of treatment. No significant differences were detected in any experimental times at the 95% confidence interval (*p* > 0.05). Plots are mean ± SE (*n* = 3).

While patterns were identified for many different parameters associated with photosynthetic activity, the most significant were those associated with the electron transport rate (ETR) and non-photochemical quenching (NPQ). In the case of the maximum quantum yield (*F_v_/F_m_*), there were no significant differences between treatments (*p* < 0.05, Figure 2, Table S2).

**Figure 3:**
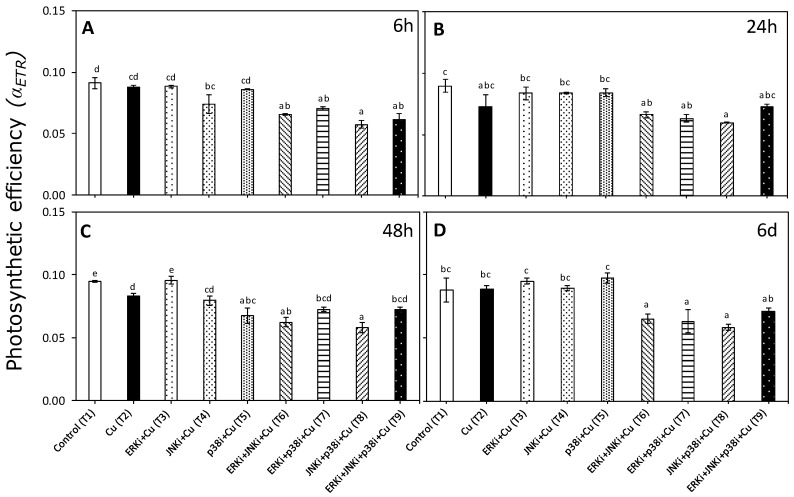
Photosynthetic efficiency (*αETR*) in *U. compressa* under copper and/or exposure to MAPK inhibitors. Treatments: T1—control only with seawater; T2—copper only exposure as 10 µM CuSO_4_ (Cu); T3—copper + 5 µM MAPK ERK inhibitor PD98059 (Cu + ERKi); T4—copper + 5 µM MAPK JNK inhibitor SP600125 (Cu + JNKi); T5—copper + MAPK p38 inhibitor SB203580 (Cu + p38i); T6—Cu + ERKi + JNKi; T7—Cu + ERKi + p38i; T8—Cu + p38i + JNKi; and T9—Cu + ERKi + JNKi + p38i. Samples were analyzed after (**A**) 6 h, (**B**) 24 h, (**C**) 48 h, and (**D**) 6 d of treatment. Different letters within each graph represent significant differences at the 95% confidence interval (*p* < 0.05). Plots are mean ± SE (*n* = 3).

There was a significant interaction between time and treatment factors for the photosynthetic efficiency (α_ETR_) data (*p* < 0.05, Table S2). Similar trends were observed in α_ETR_ at different experimental time periods. For most, there were significant differences in the α_ETR_ between controls (T1) and exposure to 10 µM copper (T2), although at 48 h, the α_ETR_ was significantly lower in the copper treatment than controls (Figure 3). The copper/MAPK inhibitor-treated material at all measurement periods tended to have lower α_ETR_ levels in all combinations than copper alone, although these differences were only significant at 6 h and 6 d (except for T9 after 6 d) (Figure 3A,B). There were no clear trends between treatments with combinations of inhibitors under copper excess at any measurement period, nor between treatments with single MAPK inhibitors and copper (Figure 3A–D).

**Figure 4:**
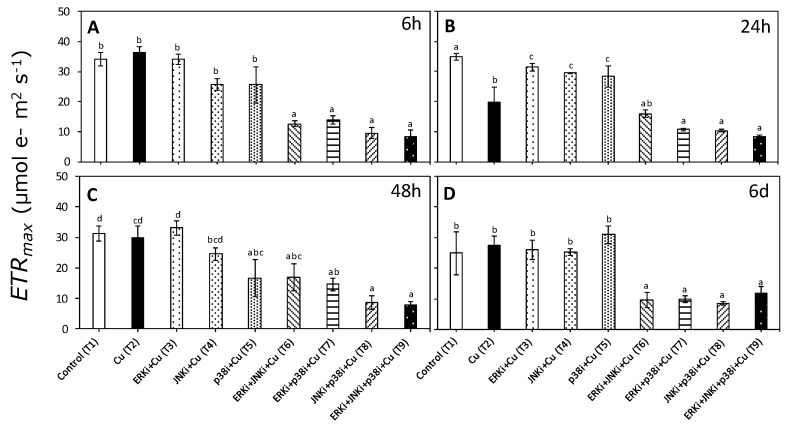
Maximal electron transport rate (ETR_max_) in *U. compressa* under copper and/or exposure to MAPK inhibitors. Treatments: T1—control only with seawater; T2—copper only exposure as 10 µM CuSO_4_ (Cu); T3—copper + 5 µM MAPK ERK inhibitor PD98059 (Cu + ERKi); T4—copper + 5 µM MAPK JNK inhibitor SP600125 (Cu + JNKi); T5—copper + MAPK p38 inhibitor SB203580 (Cu + p38i); T6—Cu + ERKi + JNKi; T7—Cu + ERKi + p38i; T8—Cu + p38i + JNKi; and T9)—Cu + ERKi + JNKi + p38i. Samples were analyzed after (**A**) 6 h, (**B**) 24 h, (**C**) 48 h, and (**D**) 6 d of treatment. Different letters within each graph represent significant differences at the 95% confidence interval (*p* < 0.05). Plots are mean ± SE (*n* = 3).

**Figure 5:**
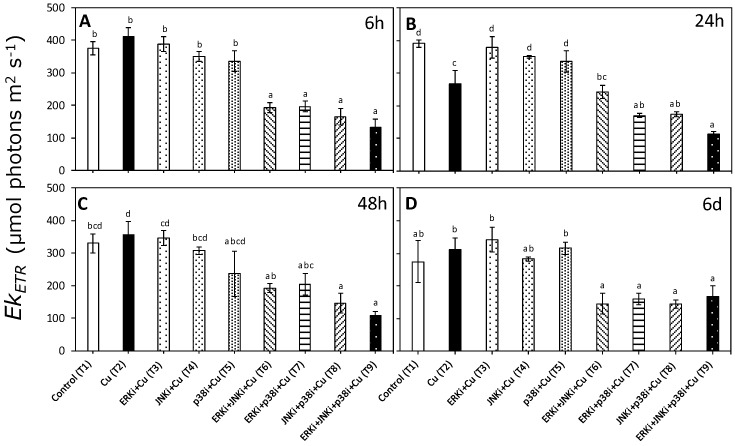
Saturation of the irradiance of ETR (EkETR) in *U. compressa* under copper and/or exposure to MAPK inhibitors. Treatments: T1—control only with seawater; T2—copper only exposure as 10 µM CuSO_4_ (Cu); T3—copper + 5 µM MAPK ERK inhibitor PD98059 (Cu + ERKi); T4—copper + 5 µM MAPK JNK inhibitor SP600125 (Cu + JNKi); T5—copper + MAPK p38 inhibitor SB203580 (Cu + p38i); T6—Cu + ERKi + JNKi; T7—Cu + ERKi + p38i; T8—Cu + p38i + JNKi; and T9)—Cu + ERKi + JNKi + p38i. Samples were analyzed after (**A**) 6 h, (**B**) 24 h, (**C**) 48 h, and (**D**) 6 d of treatment. Different letters within each graph represent significant differences at the 95% confidence interval (*p* < 0.05). Plots are mean ± SE (*n* = 3).

Patterns in ETR_max_ and Ek_ETR_ were similar to those observed for α_ETR_ with a significant interaction between treatments and times (*p* < 0.05, Table S2). There were no significant differences in ETR_max_ and Ek_ETR_ between control (T1) and copper exposure (T2) at any time period, except at 24 h (Figure 4B and Figure 5B, respectively). No clear trends were apparent between treatments combining MAPK inhibitors with copper (T6–T9) for any time period, although values of ETR_max_ and Ek_ETR_ were always lower than when exposed only to copper (T2) (Figure 4 and Figure 5).

**Figure 6:**
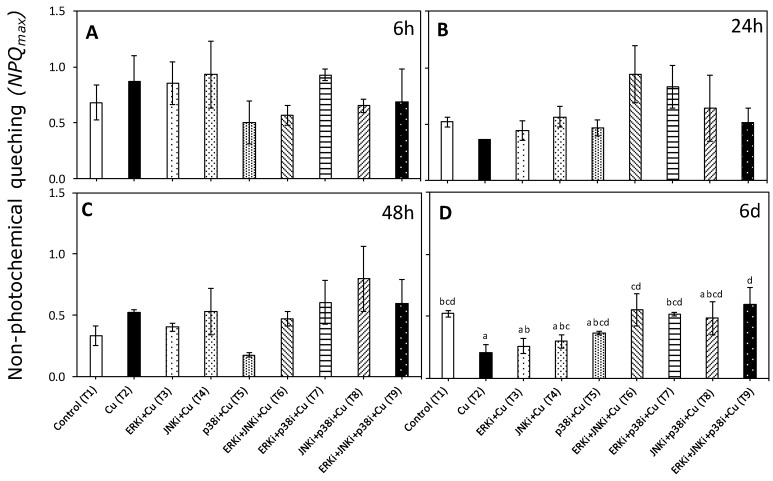
Maximal non-photochemical quenching (*NPQ_max_*) in *U. compressa* under copper and/or exposure to MAPK inhibitors. Treatments: T1—control only with seawater; T2—copper only exposure as 10 µM CuSO_4_ (Cu); T3—copper + 5 µM MAPK Extracellular Signal Regulated Kinase (ERK) inhibitor PD98059 (Cu + ERKi); T4—copper + 5 µM MAPK c-Jun *N*-terminal Kinase (JNK) inhibitor SP600125 (Cu + JNKi); T5—copper + MAPK Cytokinin Specific Binding Protein (p38) inhibitor SB203580 (Cu + p38i); T6—Cu + ERKi + JNKi; T7—Cu + ERKi + p38i; T8—Cu + p38i + JNKi; and T9—Cu + ERKi + JNKi + p38i. Samples were analyzed after (**A**) 6 h, (**B**) 24 h, (**C**) 48 h, and (**D**) 6 d of treatment. Different letters within each graph represent significant differences at the 95% confidence interval (*p* < 0.05). No significant differences were detected at experimental times of 6, 24, and 48 h (*p* > 0.05). Plots are represented as mean ± SE (*n* = 3).

There were significant differences (*p* < 0.05) in non-photochemical quenching (NPQ_max_) between certain treatments but only at day 6 (Figure 6 Table S2). Values of, NPQ_max_ were significantly lower in copper (T2) than controls, and treatments with copper combined with ERK inhibitor (T6, T7 and T9) (Figure 6D).

### 2.3. Principal Component Ordination (PCO) Analysis

**Figure 7:**
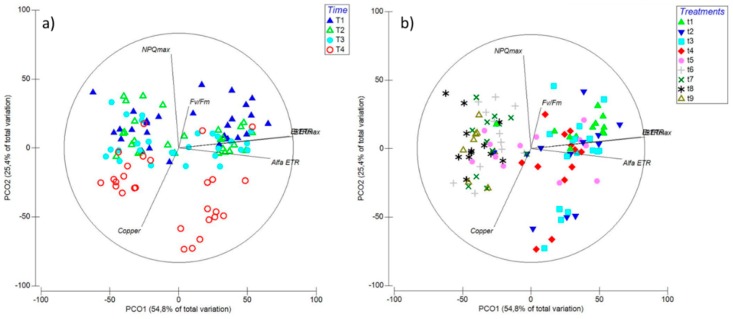
Principal Component Ordination (PCO) analysis diagrams expressed in relation to (**a**) time: T1—6 h; T2—24 h; T3—48 h; and T4—6 d; and (**b**) treatments: t1—control only with seawater; t2—copper only exposure as 10 µM CuSO_4_ (Cu); t3—copper + 5 µM MAPK ERK inhibitor PD98059 (Cu + ERKi); t4—copper + 5 µM MAPK JNK inhibitor SP600125 (Cu + JNKi); t5—copper + MAPK p38 inhibitor SB203580 (Cu + p38i); t6—Cu + ERKi + JNKi; t7—Cu + ERKi + p38i; t8—Cu + p38i + JNKi; and t9—Cu + ERKi + JNKi + p38i. Vector overlays (Spearman’s rank correlation) indicate the relationship between the PCO axes and the physiological variables, namely, intracellular copper accumulation (copper), photoinhibition (*F_v_/F_m_*), productivity (ETR_max_), efficiency (α_ETR_), and saturation of irradiance (EkETR), accompanied by higher non-photochemical quenching (NPQ_max_).

The PCO showed a positive correlation of the first axis (54.8% of total variation) with ETR_max_, Ek_ETR_, α_ETR_ and, to a lesser extent, *F_v_/F_m_*. Conversely, NPQ_max_ and intracellular copper accumulation were negatively correlated according to Spearman’s correlation (Figure 7). Time had a large effect on these factors (Figure 7a), in particular on NPQ_max_ and intracellular copper when the treatment used contained copper and two or more MAPK inhibitors (Figure 7b). In both graphical representations, the combination of the first two axes explained 80.2% of the total variation (Figure 7a,b).

## 3. Discussion

In this investigation, *Ulva compressa* was exposed to an excess copper concentration of 10 µM. Moreover, under this stress condition, the three main MAPK pathways ending in ERK, JNK, and/or p38 were blocked in order to evaluate the importance of the whole signaling pathway on metal exclusion/extrusion mechanisms and photosynthetic performance in green macroalgae under copper excess. Despite, to our knowledge, metal uptake and accumulation having important influences on the extent of toxic responses and effects, there is no published information regarding the potential roles of the MAPK pathways in these processes in eukaryotes. This is relevant given that these processes are, at least partly, transcriptionally regulated phenomena [8]. For example, it has been recorded that *Arabidopsis thaliana* mutants overexpressing the cadmium extrusion transporter AtPDR8 have a greater tolerance to cadmium excess [32]. Also, the expression of the copper-transporting ATPase HMA5 from the metallophyte *Silene vulgaris* confers increased copper tolerance in *A. thaliana* [33]. Moreover, a whole transcriptome study revealed that *Chlamydomonas reinhardtii* exposed to high silver levels overexpressed eight genes encoding hydroxyproline-rich glycoprotein components, which are constituents of the cell wall, and glycoside hydrolase, which aids in remodelling of the cell wall [34]. These findings are important, since metal-extrusion/exclusion mechanisms in macroalgae are known to involve chelation to cell wall components and energy-dependent actions of membrane transporters [9]. Although the role of the MAPK signaling pathway in activating the metal-extrusion/exclusion mechanisms in algae has not been explored, results from studies with plants provide insight into its potential participation. For instance, it was observed that several transcription factors regulating metal tolerance responses, such as C2H2-type zinc finger transcription factor (ZAT), basic region leucine zipper (bZIP), and myeloblastosis (MYB), were controlled upstream by MAPKs [8,35,36]. Wang et al. [37] observed that the MYB transcription factor OsARM1(ARSENITE-RESPONSIVE MYB1) bound to the promoters of OsLsi1, OsLsi2, and OsLsi6 that encoded and regulated the expression of key arsenic transporters. Interestingly, when *U. compressa* was exposed to copper and at least one (except JNK) MAPK pathway inhibitor, intracellular copper accumulation decreased markedly compared to when exposed to copper alone. Evidence from other photoautotrophs suggested that partial or complete inhibition of the MAPK pathways overstimulates other signaling pathways and enhances copper exclusion/extrusion. This indicated crosstalk between, for example, calcium, NO, and ROS signaling, as previously described in *U. compressa* under copper excess [9,23]. Even though we did not find other similar investigations on photoautotrophs, support for this view came from a study on human bone-marrow stroma cells (Doan et al. 2012). Inhibition of the JNK or ERK pathways induced an increase in calcium content and deposition of these cells, overstimulating bone differentiation [38]. In contrast, when blocking the p38 pathway, there were lower concentrations and deposition of calcium, followed by decreased rates of bone differentiation [38]. Given that crosstalk between calcium and MAPK signaling pathways has been described for plants [39,40], it is possible that impairment of one, with the exception of JNK, or more pathways within the MAPKs may overstimulate other signaling mechanisms, enhancing exclusion/extrusion mechanisms and decreasing total intracellular accumulation under copper excess in *U. compressa.* Furthermore, our findings suggested that only blocking JNK results in increased intracellular copper accumulation, while blocking p38 (alone or in combination) gave rise to the lowest cellular concentrations. These results suggested that JNK was the least relevant and p38 the most pertinent MAPK pathway for copper exclusion/extrusion in *U. compressa*; these findings warrant further detailed investigation.

Although reports of involvement of the MAPK pathways in photosynthesis are scarce, a connection was described for *Glycine max* (soya bean). By blocking MEKK1 (JNK pathway), a dramatic downregulation of genes was observed in the primary metabolism, including those related to photosynthesis [41]. In contrast, in the microalga *Vischeria helvetica* (Ochrophyta), synthesis of carotenoids increased when p38 and ERK (but not JNK) MAPK pathways were inhibited [42]. In other studies, no effects were found; for example, in *A. thaliana* mutants deprived of MPK4 (upstream JNK), there were no significant differences with wildtypes in terms of *F_v_/F_m_*, rETR, and NPQ [43], and in the microalga *Asterochloris erici* (chlorophyte), when the p38 and JNK pathways were inhibited, no changes in *F_v_/F_m_* were observed, even under hyperosmotic conditions [44]. Despite these mixed results, the MAPK pathways do appear to play a role in photosynthesis, although the overall contribution of the ERK, JNK, and p38 MAPK pathways in regulating photosynthetic activity in photoautotrophs is not clear and their involvement under environmental stress, such as metal pollution, is comparatively unknown.

Negative controls exposing *U. compressa* to MAPK pathway inhibitors without added copper showed no patterns in in vivo chlorophyll *a* fluorescence parameters associated with PSII compared to algae grown in seawater (see Section 4). Therefore, the observed differences in photosynthetic activity under copper excess compared with controls and those subjected to copper and inhibitors, especially when more than one MAPK was blocked, were indeed a consequence of exposure to the metal and inhibition of MAPK pathways. Since no changes were recorded for *F_v_/F_m_*, our findings suggested that no transient damage to the antenna complex was induced under different treatments [12,29]. In contrast, during most of the measurement periods, α_ETR_, ETR_max_, and EK_ETR_ under copper in combination with two or more blocked MAPK pathways were always lower than controls and when only exposed to copper. This trend was especially evident after 6 d of exposure, and behaviour was similar for algae under copper and mixed MAPK inhibitors. Rapid light curves (RLCs), from which α_ETR_, ETR_max_, and EK_ETR_ were derived, indicated that the algae exposed to a combination of blocked MAPK pathways subject to copper excess displayed diminished photosynthetic efficiency, maximal electron transport rate, and saturation of irradiance of ETR, respectively [12,26,29]. The latter is coincidental, since it is known that copper excess inhibits photosynthesis by reducing the chlorophyll content as a consequence of copper-induced iron deficiency [25]. Moreover, it is well recognized that copper stress disrupts electron transport chains in PSII, with the excess energy being transferred to oxygen, for example, via the triplet state of chlorophyll, thereby inducing overproduction of singlet oxygen (^1^O_2_), a highly oxidizing ROS [9,43]. Excess energy in chloroplasts is mainly counteracted by the synthesis of carotenoids, among which xanthophylls are the most important [43,45,46]. Thermal dissipation can be measured as non-photochemical PSII fluorescence quenching (NPQ), which is triggered by the trans-thylakoidal proton gradient (ΔpH) and zeaxanthin (ZEA) synthesis through the xanthophyll cycle [29]. Our results from day 6 revealed that when two or more MAPK pathways were blocked under copper (except JNK and p38), NPQ_max_ values were higher than for algae subjected only to copper, reaching control values. Although there is limited related information, it was recently discovered in *A. thaliana* that upstream activation of p38, MPK3, and MPK6 induces downregulation of different genes associated with photosynthesis, among which are those associated with light reactions, plastid organization, responses to light stimulus, electron transport chains, metabolism of porphyrin-associated compounds, tetrapyrrole metabolic processes, light harvesting, and pigment biosynthesis [47]. Our results suggested that, in *U. compressa*, blocking two pathways or the whole MAPK pathway under copper excess caused a decrease in photosynthesis and excess energy transfer, which may have been counteracted by increased energy dissipation through heat. Furthermore, our data supported the idea that there was crosstalk between ERK, JNK, and p38 pathways under copper stress, and that photosynthetic activity could be maintained even when one pathway within the MAPK group was impaired. However, if more than one pathway was blocked then photosynthetic performance was compromised, demonstrating the importance of the whole MAPK pathway to ensure balanced photosynthetic activity in *U. compressa* under environmental stress.

Considering the whole dataset presented in this investigation through PCO analysis, the differences in the various measured parameters were mainly induced after 6 days of exposure, by which time the highest correlations were represented by α_ETR_, ETR_max_, and EK_ETR_. Furthermore, most of the significant differences were induced under copper when blocking two or more MAPK pathways.

In this investigation, we identified that the whole MAPK pathway plays an important role in the physiological responses associated with metal accumulation and photosynthetic activity in *Ulva compressa* under copper stress. In particular, blocking one or more of the MAPK pathways, with the exception of JNK, decreased intracellular copper accumulation, which may have been a consequence of enhanced metal exclusion/extrusion mechanisms through crosstalk and activation of other cellular signaling pathways (e.g., calcium, NO, ROS). Moreover, by inhibiting most of the MAPK pathways (at least two out of ERK, JNK, and p38), photosynthetic activity was impaired, supporting the idea of potential crosstalk between the different studied MAPK pathways. To confirm most of these hypotheses, a whole transcriptome representation of the blocked MAPK pathways under copper stress in *U. compressa* is currently being explored in our ongoing investigations. To complement this article, in a parallel manuscript [1], we explore the roles of the ERK, JNK, and p38 MAPK pathways in the copper-detoxification mechanisms associated with antioxidant metabolism and synthesis of metal chelators in *U. compressa*.

## 4. Materials and Methods

### 4.1. Sample Collection and Culture Environment Conditions

All samples of *Ulva compressa* L. (Chlorophyta, Ulvaceae) were collected from Cachagua beach, Valparaíso, Chile (32°34′59″S; 71°26′16″W), a location with no history of metal pollution. Adult individuals were sampled from the intertidal zone, placed in plastic bags with seawater, and transported to the laboratory in a cooler. Algal material was successively washed with filtered seawater (2 µm), and stored inside plastic containers in a culture chamber with constant aeration at 16 °C, with a photoperiod cycle of 12:12 (day/night); daylight conditions corresponded to 160 µmol·m^2^·s^−1^ photosynthetic active radiation (PAR). *Ulva compressa* was acclimated for 48 h prior to the start of the experiment.

### 4.2. Copper and MAPK Inhibitor Treatments

Following the acclimation period, 5 g fresh weight (FW) samples of macroalgae were placed in 300 mL plastic containers with 200 mL of 2 µm filtered and autoclaved seawater. A chronic copper concentration of 10 µM was chosen, based on previously obtained physiological viability measurements [48]. There was a total of nine treatments, with three replicates each: T1—control with only seawater; T2—chronic copper exposure at 10 µM CuSO_4_ (Sigma-Aldrich, St. Louis, MO, USA) (Cu); T3—Cu + 5 µM MAPK ERK inhibitor PD98059 (Tocris Bioscience, St. Louis, MO, USA)) (Cu + ERKi); T4—Cu + 5 µM MAPK JNK inhibitor SP600125 (Tocris Bioscience) (Cu + JNKi); T5—Cu + MAPK p38 inhibitor SB203580 (Tocris Bioscience, St. Louis, MO, USA)) (Cu + p38i); T6—Cu + ERKi + JNKi; T7—Cu + ERKi + p38i; T8—Cu + p38i + JNKi; and T9—Cu + ERKi + JNKi + p38i. Negative controls were included, comprising seawater and ERKi, JNKi, or p38; there were no significant differences in photosynthetic activity between these treatments (see Supplementary Figures) nor in gene expression (see Supplementary Figure in Rodríguez-Rojas et al.) [1]. The inhibitors were initially developed to target human ERK, JNK, and p38 MAPKs, although they were subsequently used successfully in studies on invertebrates [49,50], vertebrates [51,52], plants [53,54,55], and algae [56,57]. Moreover, ERK, JNK, and p38 mammalian-like MAPKs were identified in macroalgae species, including the ulvophytes *Ulva rigida* and *Chaetomorpha aerea* [58,59]. ERK-encoding genes showed high degrees of homology between eukaryotes, among which included the microalga *Dunaliella viridis*, the plant *Arapidopsis thaliana*, the amoeba *Dictyostelium discoideum*, the protozoan *Plasmodium falciparum,* the mammal *Mus musculus*, and humans [60]; thus, it was not unexpected that human-developed inhibitors were also effective in a wide range of eukaryotes due to the highly conserved nature of MAPKs.

Exposure to all treatments took 6 d, with measurements of copper accumulation and photosynthetic activity taken at 6 h, 24 h, 48 h, and 6 d. Treatment solutions were changed every 48 h to avoid nutrient, copper, and inhibitor limitations.

### 4.3. Intracellular Copper Accumulation

At each measurement point, 1 g fresh weight (FW) of macroalgae was collected and washed twice for 15 min with 10 mM ethylene diamine tetra acetic acid (EDTA), as described in Roncarati et al. [19]; the latter allowed for the extraction of metal bound to cell walls and intracellular spaces and provided a measure of only intracellular copper accumulation. Macroalgae were then dried until a constant weight in an oven at 60 °C. Samples were digested with modifications to the protocol of US EPA 3052. Initially, samples were pre-digested overnight at room temperature in teflon vials with 6 mL of 65% nitric acid (Merck^®^) and 2 mL of 30% hydrogen peroxide (Merck^®^), then placed in a microwave oven (Milestone^®^, model Ethos Easy). Samples were digested according to the following cycle: 10 min at 200 °C (1800 W power) and then 20 min at 200 °C (1800 W power). Once the digestion process was completed, samples were cooled at room temperature for 30 min. Digests were then brought to 25 mL with ultrapure (Milli Q; Adrona^®^, model CB-1903, Riga, Letonia) water and stored in the dark at room temperature for analyses. Copper was measured using inductively coupled plasma optical emission spectrometry (ICP-OES; Perkin-Elmer^®^, model ICP Optima 2000 DV, Wellesley, MA, USA). To ensure precision and accuracy of the results, the methodology was also applied to certified reference material (CRM) of sea lettuce (*U. lactuca*; BCR-279). There was significant agreement between the measured and certified levels, with values ranging between 92% and 105% (*p* > 0.05).

### 4.4. Photosynthesis and Energy Dissipation through In Vivo Chlorophyll a Fluorescence

In vivo chlorophyll *a* fluorescence associated with photosystem II (PSII) was determined using a portable fluorometer Junior PAM fluorometer with WinControl-3.2 software (Walz GmbH, Effeltrich, Germany). Samples of *U. compressa* were collected at each measurement point (6 h, 24 h, 48 h, and 6 days) and placed in 10 mL incubation chambers to obtain rapid light curves (RLC) for each treatment (three RLCs per treatment). RLCs represented the saturation characteristics of PSII electron transport and overall photosynthetic performance [26,29].

### 4.5. Maximum Quantum Yield of PSII (Fv/Fm).

In order to conduct RLCs, *F_o_* (basal fluorescence from fully oxidized reaction centers of PSII) and *F_m_* (maximal fluorescence from fully reduced PSII reaction center), were measured after 15 min in darkness. The Maximal quantum yield (*F_v_/F_m_*), was derived from these parameters, with *F_v_* being the difference between *F_m_* and *Fo* [28].

*Electron transport rate (ETR)* is an indicator of productivity and photosynthetic capacity [26,29]. ETRs were determined after 20 s of the exposure period under twelve incremental irradiances of blue light (E1 = 25, E2 = 45, E3 = 66, E4 = 90, E5 = 125, E6 = 190, E7 = 285, E8 = 420, E9 = 625, E10 = 845, E11 = 1150, and E12 = 1500 µmol m^2^·s^−1^).

The ETR was calculated according to Schreiber et al. [28]:

*ETR* (μmol e^−^·m^−2^·s^−1^) =Δ*F*/*F_m_*’ × *E* × *A* × *FII* (1)

where Δ*F/F_m_’* is the effective quantum yield, Δ*F = (F_m_’ − F_t_)*, *F_t_* is the intrinsic fluorescence of alga incubated in light, and *F_m_’* is the maximal fluorescence reached after a saturation pulse of the alga incubated in light, E is the incident PAR irradiance expressed in μmol photons m^−2^ s^−1^, A is the thallus absorbance as a fraction of incident irradiance that is absorbed by the alga, and FII is the fraction of chlorophyll related to PSII (400–700 nm), being 0.5 in green macroalgae [26].

The photosynthetic parameters, i.e., the *maximal electron transport rate* (ETR_max_, estimate of maximal photosynthetic capacity) and the *photosynthetic efficiency* (α_ETR_, estimate of photosynthetic efficiency in the RLC), were obtained from the tangential model function reported by Eilers and Peeters [61]. The irradiance at which ETR (Ek_ETR_) was saturated was calculated from the intercept between ETR_max_ and α_ETR_.

*Non-photochemical quenching (NPQ)*, as an indicator of energy dissipation and photoprotection, was also obtained from the RLC. The NPQ was calculated according to Schreiber et al. [28] as:

*NPQ* = (*F_m_*/*F_m_*’)/*F_m_*’ (2)

*Maximal non-photochemical quenching (NPQ_max_)* and the initial slope of NPQ versus irradiance curves (α_NPQ_) were obtained from the tangential model function of NPQ vs irradiance function.

### 4.6. Statistical Analyses

Effects on physiological variables and intracellular copper content were analyzed using ANOVA with the software SPSS v.21 (IBM, Armonk, NY, USA). This test was performed per time treatment (one-way) as a fixed factor for all variables (mean ± SE, *n* = 3). Each time period (6 h, 24 h, 48 h, and 6 days) included a treatment factor with nine levels, T1, T2, T3, T4, T5, T6, T7, T8, and T9, with a level of probability applied in the statistical analyses at *p* < 0.05. Homogeneity and homoscedasticity were tested using Cochran tests and by visual inspection of the residuals. In the case of significant ANOVAs, a posteriori Student Newman Keuls tests (SNK) were performed to evaluate the differences between groups.

A multi-dimensional scaling (MDS) or Principal Coordinates Ordination (PCO) analysis [62] was performed to detect patterns among parameters on the basis of Euclidean distance using PERMANOVA + PRIMER6 package. PCO analyses were conducted for *F_v_/F_m_*, α_ETR_, ETR_max_, Ek_ETR_, NPQ_max_ and intracellular copper accumulation for each treatment and time period. This procedure was an equivalent ordination to a principal component analysis (PCA), calculating the percentage variation explained by each of the axes in the multidimensional scale. The overlay of the vectors onto the PCO was performed using Spearman’s correlation [63].

## Supplementary Materials

Supplementary materials can be found at https://www.mdpi.com/1422-0067/20/18/4547/s1.

## Abbreviations

MDPI	Multidisciplinary Digital Publishing Institute
MAPK	mitogen activated protein kinases
ERK	Extracellular Signal Regulated Kinases
JNK	c-Jun *N*-terminal Kinases
p38	Cytokinin Specific Binding Protein
ROS	reactive oxygen species
F_v_/F_m_	Maximal quantum yield of photosystem II
ETR_max_	maximal electron transport rate of photosynthesis
EkETR	Saturation of the irradiance of electron transport rate
α_ETR_	photosynthetic efficiency
NPQ_max_	maximal non-photochemical quenching
