# Supplementary material for: MAPK Pathway under Chronic Copper Excess in Green Macroalgae (Chlorophyta): Influence on Metal Exclusion/Extrusion Mechanisms and Photosynthesis
Source: Int J Mol Sci. 2019 Sep 13;20(18):4547. doi: 10.3390/ijms20184547 (PMC6769437; doi:10.3390/ijms20184547)
Supplement: Supplementary file 1 [file ijms-20-04547-s001.pdf]

Table Supplementary 1: ANOVA results after experiment testing for the effect of treatments on the copper accumulation, maximal quantum yield (F_v/F_m), photosynthetic efficiency (α_{ETR}), maximal electron transport rate (ETR_{max}), irradiance of saturation (Ek_{ETR}) and Maximal non-photochemical quenching (NPQ_{max}) of *Ulva compressa*. p<0.05**

			Ulva Compressa			
			df	***MS***	***F***	***P***
Copper	*Time 1*	*Treatments*	8	**1697.71**	**3205.46**	**
		Res	18	0.53		
	Time 2	*Treatments*	8	**2264.59**	**3952.9**	**
		Res	18	0.57		
	Time 3	*Treatments*	8	**3092.48**	**15272.6**	**
		Res	18	0.20		
	Time 4	*Treatments*	8	**64710.83**	**20829**	**
		Res	18	3.11		
F_v/F_m	*Time 1*	*Treatments*	8	0.005	1.61	0.19
		Res	18	0.003		
	Time 2	*Treatments*	8	0.005	1.48	0.23
		Res	18	0.003		
	Time 3	*Treatments*	8	0.009	2.05	0.10
		Res	18	0.004		
	Time 4	*Treatments*	8	0.004	1.24	0.33
		Res	18	0.003		
α_{ETR}	*Time 1*	*Treatments*	8	**0.00050**	**12.95**	**
		Res	18	0.00004		
	Time 2	*Treatments*	8	**0.00034**	**5.54**	**
		Res	18	0.00006		
	Time 3	*Treatments*	8	**0.00053**	**15.58**	**
		Res	18	0.00003		
	Time 4	*Treatments*	8	**0.00068**	**8.62**	**
		Res	18	0.00008		
ETR_{max}	*Time 1*	*Treatments*	8	**383.17**	**17.66**	**
		Res	18	21.70		
	Time 2	*Treatments*	8	**311.62**	**22.80**	**
		Res	18	13.66		
	Time 3	*Treatments*	8	**276.20**	**8.55**	**
		Res	18	32.29		
	Time 4	*Treatments*	8	**250.19**	**8.52**	**
		Res	18	29.35		
Ek_{ETR}	*Time 1*	*Treatments*	8	**36065.34**	**22.59**	**
		Res	18	1596.52		
	Time 2	*Treatments*	8	**30551.81**	**20.29**	**
		Res	18	1505.43		
	Time 3	*Treatments*	8	**24995.46**	**7.30**	**
		Res	18	3422.15		
	Time 4	*Treatments*	8	**20141.01**	**6.15**	**
		Res	18	3272.65		
NPQ_{max}	*Time 1*	*Treatments*	8	0.076	0.68	0.71
		Res	18	0.113		
	Time 2	*Treatments*	8	0.107	1.42	0.25
		Res	18	0.075		
	Time 3	*Treatments*	8	0.094	1.50	0.23
		Res	18	0.063		
	Time 4	*Treatments*	8	**0.060**	**2.78**	**
		Res	18	0.021		

Res: Residual

Table Supplementary 2 : ANOVA results after experiment testing for the effect of treatments with *U. compressa;* in control conditions, inhibitor ERK, inhibitor JNK and inhibitor p38. In response to physiological variables: maximal quantum yield (F_v/F_m), photosynthetic efficiency (α_{ETR}), maximal electron transport rate (ETR_{max}), irradiance of saturation (Ek_{ETR}) and Maximal non-photochemical quenching (NPQ_{max}) of *Ulva compressa*. p<0.05**

			Ulva Compressa			
			df	***MS***	***F***	***P***
F_v/F_m	*Time 1*	*Treatments*	4	0.0009	0.64	0.64
		Res	10	0.0014		
	Time 2	*Treatments*	4	0.0122	1.45	0.29
		Res	10	0.0084		
	Time 3	*Treatments*	4	0.0021	1.13	0.40
		Res	10	0.0019		
	Time 4	*Treatments*	4	0.0049	1.00	0.45
		Res	10	0.0049		
α_{ETR}	*Time 1*	*Treatments*	4	0.00008	1.30	0.33
		Res	10	0.00006		
	Time 2	*Treatments*	4	0.00016	0.79	0.55
		Res	10	0.00020		
	Time 3	*Treatments*	4	0.00005	3.02	0.07
		Res	10	0.00002		
	Time 4	*Treatments*	4	0.00012	1.14	0.39
		Res	10	0.000		
ETR_{max}	*Time 1*	*Treatments*	4	101.9	4.37	0.03
		Res	10	23.3		
	Time 2	*Treatments*	4	113.2	1.89	0.19
		Res	10	59.8		
	Time 3	*Treatments*	4	24.1	1.17	0.38
		Res	10	20.6		
	Time 4	*Treatments*	4	20.7	0.43	0.78
		Res	10	48.2		
Ek_{ETR}	*Time 1*	*Treatments*	4	10425.5	3.25	0.06
		Res	10	3204.7		
	Time 2	*Treatments*	4	8777.1	1.29	0.34
		Res	10	6814.9		
	Time 3	*Treatments*	4	2983.2	1.17	0.38
		Res	10	2552.8		
	Time 4	*Treatments*	4	2544.8	0.52	0.72
		Res	10	4855.5		
NPQ_{max}	*Time 1*	*Treatments*	4	0.358	1.47	0.28
		Res	10	0.243		
	Time 2	*Treatments*	4	0.105	1.03	0.44
		Res	10	0.102		
	Time 3	*Treatments*	4	0.025	2.47	0.11
		Res	10	0.010		
	Time 4	*Treatments*	4	2614293.6	1.00	0.45
		Res	10	2614216.2		

Res: Residual

Supplementary figures

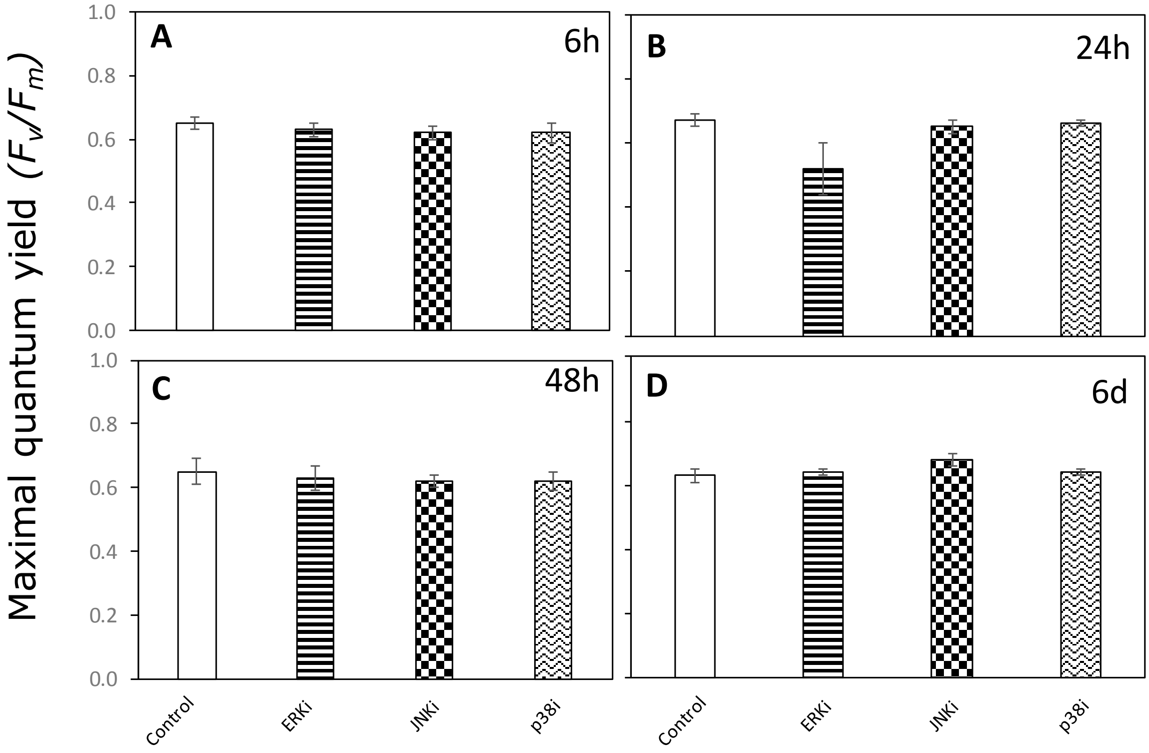

Supplementary Figure 1.

Supplementary figure 1. Maximum quantum yield of PSII (*Fv/Fm*) in *U. compressa* exposed to control conditions and MAPK inhibitors. Treatments consisted in: T1) Control conditions (no copper added); T2) 5 µM MAPK ERK inhibitor PD98059 in seawater (ERKi) ; T3) 5 µM MAPK JNK inhibitor SP600125 in seawater (JNKi); T4) MAPK p38 inhibitor SB203580 in seawater (p38i). Samples were analyzed after 6 h (A), 24 h (B), 48 h (C) and 6 d (D) treatments. Treatments did not present significant differences at 95% confidence interval ($p > 0.05$). Plots are represented as mean ± SE ($n = 3$).

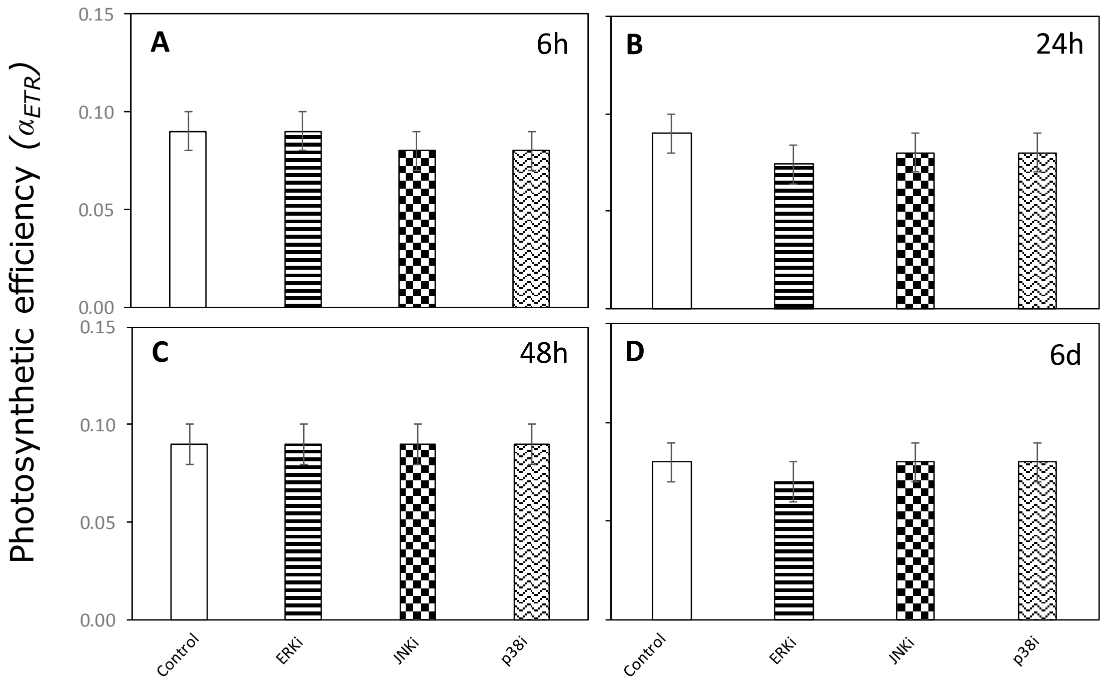

Supplementary Figure 2.

Supplementary figure 2. Photosynthetic efficiency (*αETR*) in *U. compressa* exposed to control conditions and MAPK inhibitors. Treatments consisted in: T1) Control conditions (no copper added); T2) 5 µM MAPK ERK inhibitor PD98059 in seawater (ERKi) ; T3) 5 µM MAPK JNK inhibitor SP600125 in seawater (JNKi); T4) MAPK p38 inhibitor SB203580 in seawater (p38i). Samples were analyzed after 6 h (A), 24 h (B), 48 h (C) and 6 d (D) treatments. Treatments did not present significant differences at 95% confidence interval ($p > 0.05$). Plots are represented as mean ± SE ($n = 3$).

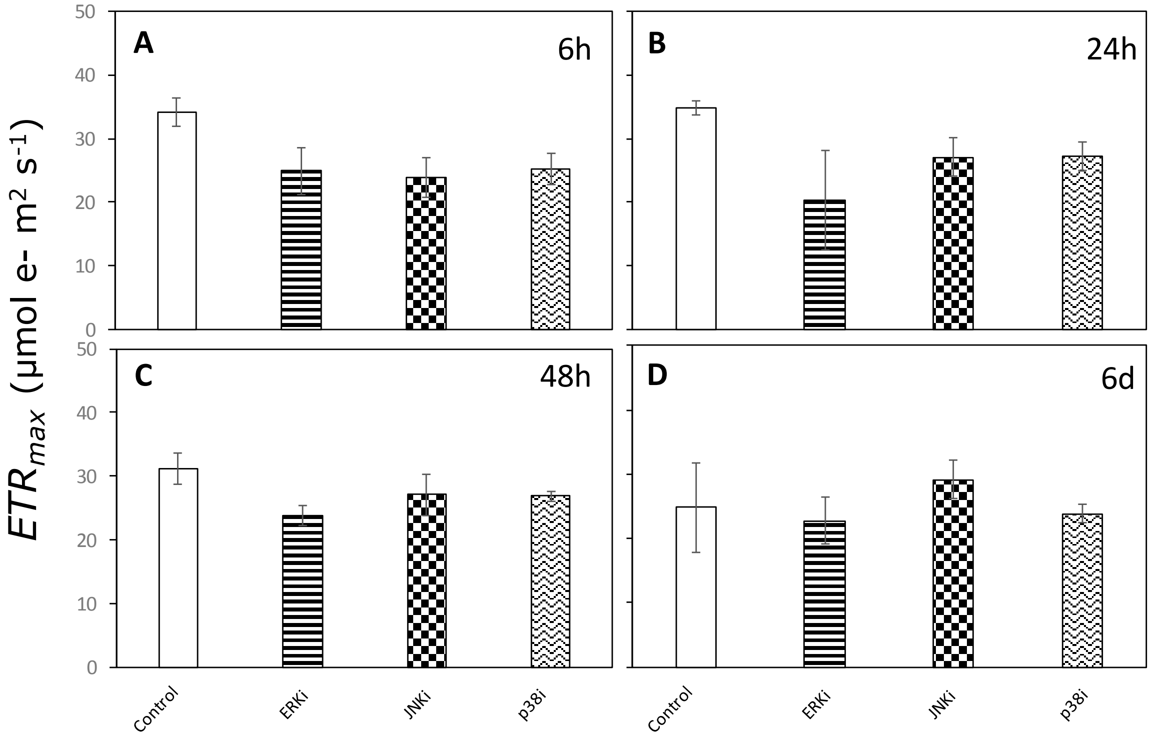

Supplementary Figure 3.

Supplementary figure 3. Maximal electron transport rate (ETR_{max}) in *U. compressa* exposed to control conditions and MAPK inhibitors. Treatments consisted in: T1) Control conditions (no copper added); T2) 5 µM MAPK ERK inhibitor PD98059 in seawater (ERKi) ; T3) 5 µM MAPK JNK inhibitor SP600125 in seawater (JNKi); T4) MAPK p38 inhibitor SB203580 in seawater (p38i). Samples were analyzed after 6 h (A), 24 h (B), 48 h (C) and 6 d (D) treatments. Treatments did not present significant differences at 95% confidence interval ($p > 0.05$). Plots are represented as mean ± SE ($n = 3$).

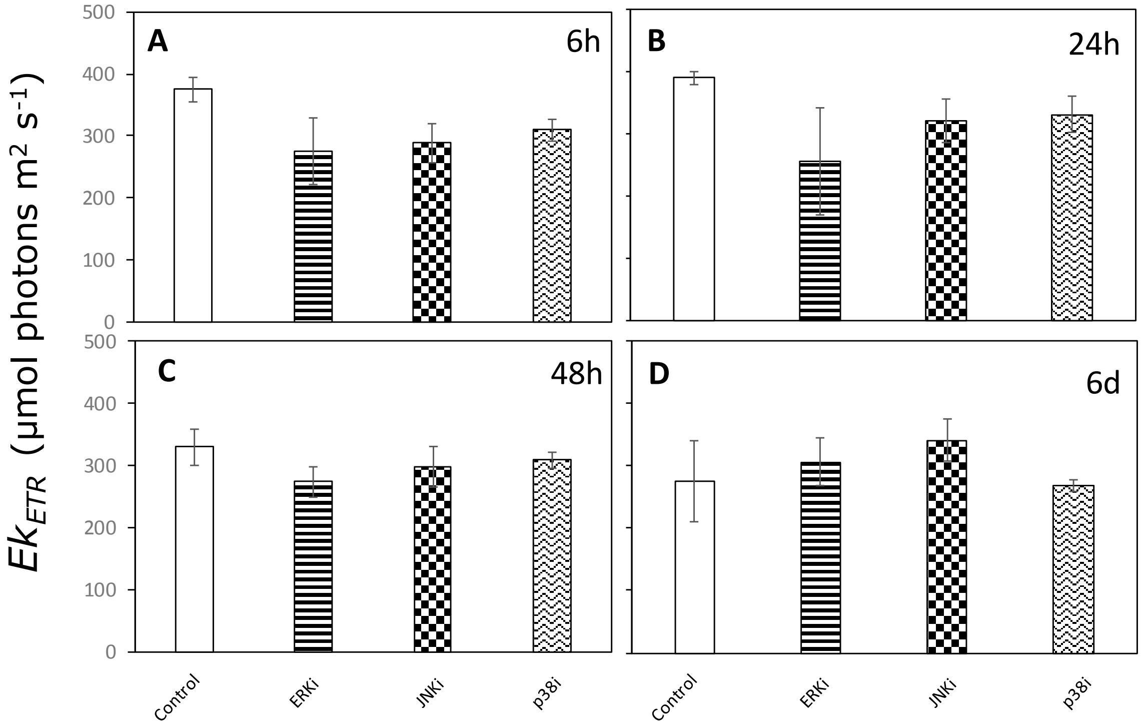

Supplementary Figure 4.

Supplementary figure 4. Saturation of the irradiance of ETR (Ek_{ETR}) in *U. compressa* exposed to control conditions and MAPK inhibitors. Treatments consisted in: T1) Control conditions (no copper added); T2) 5 µM MAPK ERK inhibitor PD98059 in seawater (ERKi) ; T3) 5 µM MAPK JNK inhibitor SP600125 in seawater (JNKi); T4) MAPK p38 inhibitor SB203580 in seawater (p38i). Samples were analyzed after 6 h (A), 24 h (B), 48 h (C) and 6 d (D) treatments. Treatments did not present significant differences at 95% confidence interval ($p > 0.05$). Plots are represented as mean ± SE ($n = 3$).

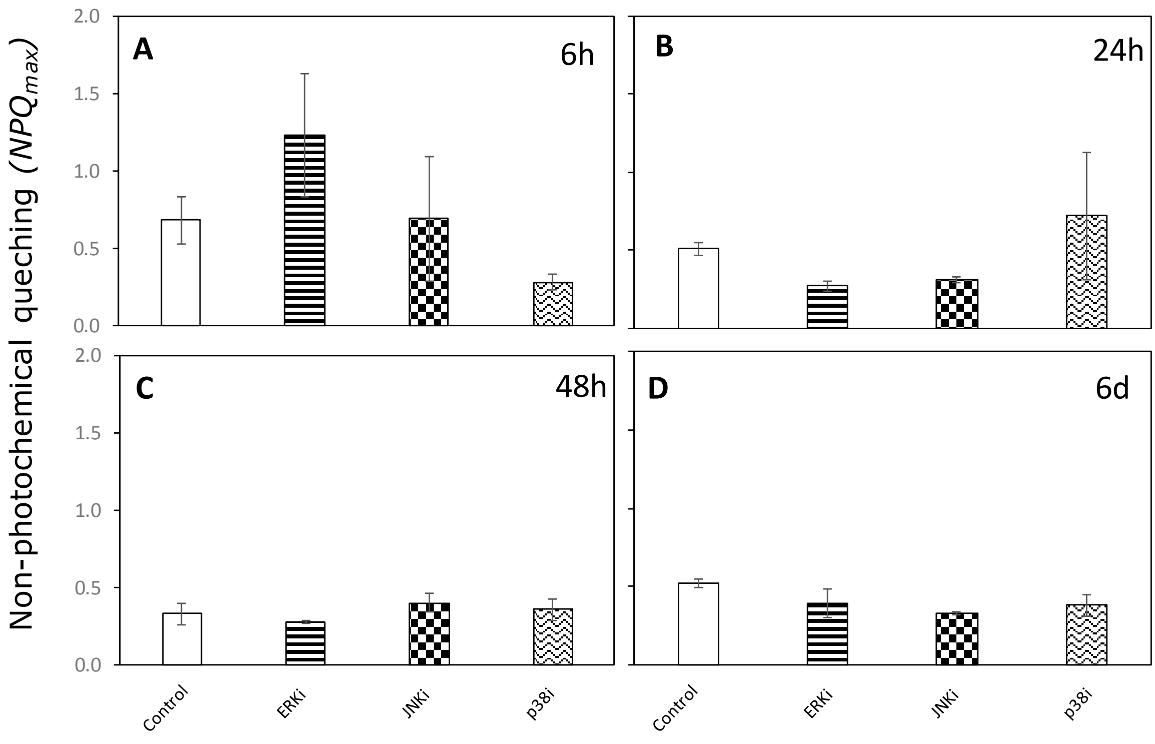

Supplementary Figure 5.

Supplementary figure 5. Maximal non-photochemical quenching (NPQ_{max}) in *U. compressa* exposed to control conditions and MAPK inhibitors. Treatments consisted in: T1) Control conditions (no copper added); T2) 5 μM MAPK ERK inhibitor PD98059 in seawater (ERKi) ; T3) 5 μM MAPK JNK inhibitor SP600125 in seawater (JNKi); T4) MAPK p38 inhibitor SB203580 in seawater (p38i). Samples were analyzed after 6 h (A), 24 h (B), 48 h (C) and 6 d (D) treatments. Treatments did not present significant differences at 95% confidence interval ($p > 0.05$). Plots are represented as mean ± SE ($n = 3$).
